# Liver Stiffness Is a Predictor of Rebleeding in Patients With Hepatitis B-Related Cirrhosis: A Real-World Cohort Study

**DOI:** 10.3389/fmed.2021.690825

**Published:** 2021-07-28

**Authors:** Linxiang Liu, Yuan Nie, Yue Zhang, Qi Liu, Xuan Zhu

**Affiliations:** Department of Gastroenterology, The First Affiliated Hospital of Nanchang University, Nanchang, China

**Keywords:** cirrhosis complications, hepatitis B virus, liver stiffness, non-invasive fibrosis score, rebleeding

## Abstract

**Background:** Esophageal vein rebleeding is a life-threatening complication of liver cirrhosis. However, the role of non-invasive methods that were developed to evaluate the severity of chronic liver disease, especially in rebleeding, remains unclear.

**Aims:** To evaluate the performance of liver stiffness and non-invasive fibrosis scores in predicting esophageal vein rebleeding in hepatitis B virus (HBV) cirrhotic patients.

**Methods:** A prospective analysis of 194 HBV patients between 2017 and 2021 was performed. Receiver operating characteristic (ROC) curves and time-dependent ROC curves were used to assess the power for predicting rebleeding with non-invasive fibrosis score and liver stiffness.

**Results:** During the median follow-up time of 68.28 weeks, 55 patients experienced rebleeding. In the entire cohort, the area under the ROC curve for liver stiffness measurement (LSM) predicting for rebleeding was 0.837, with a cut-off value of 17.79 kPa, and the time-dependent ROC curve also showed stable prediction performance of LSM. The predictive ability of the non-invasive fibrosis score was less than that of LSM, and there were statistical differences. Moreover, patients using non-selective beta-blockers and HBV DNA-negative patients experienced significantly reduced rebleeding.

**Conclusions:** Compared with non-invasive fibrosis scores, LSM can more simply and accurately predict rebleeding events of hepatitis B liver cirrhosis.

## Introduction

Liver cirrhosis is caused by various injury mechanisms which can induce liver necrosis and fibrosis. It is considered not a single disease entity but a disease that can be subdivided into different clinical prognostic stages (1). Esophageal variceal bleeding (EVB) is a common complication of liver cirrhosis, and it can also be a life-threatening complication due to high morbidity and high mortality (2). Despite improvements in the efficacy of endoscopic, pharmacologic, surgical, and radiologic techniques, the 6-week mortality and total mortality after bleeding are 17.5 and 33.5%, respectively (3). Not only is the mortality rate of the first esophageal venous bleeding high, but the 6-week rebleed rate is up to 60% in patients who have not undergone secondary prevention patients (4). Therefore, the occurrence of rebleeding events has received increasing attention in cirrhosis patients.

Recent studies pooling available evidence have demonstrated that the severity of liver fibrosis, especially the presence of advanced fibrosis defined as stage F3 or F4 fibrosis, is the main driver of prognosis in cirrhosis and the main risk factor for developing not only liver-related events but also extrahepatic complications (5–7). In this context, there is a good correlation between the non-invasive fibrosis score and the degree of liver fibrosis (5, 8–10), and the degree of liver fibrosis was correlated with the degree of portal hypertension. However, the use of the fibrosis score to predict the occurrence of complications of liver cirrhosis, especially the rebleeding of the esophageal vein of liver cirrhosis, is a major unmet need.

Liver stiffness measurement (LSM) is a widely used non-invasive tool for the diagnosis of liver fibrosis and has high accuracy (11), and combined platelets are also used to identify patients at high risk for esophageal varices without the need for endoscopic screening (12). Previous studies have demonstrated that liver stiffness can reflect the prognosis of patients with liver cirrhosis because it can indirectly reflect portal hypertension (11, 13). Liver stiffness measured using transient elastography (TE) has been validated as a prognostic quantitative marker for the occurrence of liver-related complications, survival without liver-related death, and overall survival (6, 14–16). However, LSM has not been well-verified in the esophageal variceal rebleeding, which is a critical event.

Chronic hepatitis B (CHB) is a major health burden, with an estimated 240 million chronic carriers of the hepatitis B virus (HBV) surface antigen (HBsAg) worldwide, resulting in 815,000 people dying annually due to its complications (17, 18). TE and fibrosis scores currently focus on the accuracy of the pathological classification of liver cirrhosis. However, there are few studies predicting the complications of esophageal venous rebleeding in liver cirrhosis, which makes it difficult for clinicians to accurately and rapidly evaluate such patients and increases the burden of public health. To address this limitation, our study aims to evaluate non-invasive serological indices, namely, the AST to Platelet Ratio Index (APRI), Fibrosis-4 score (FIB-4), King's College Criteria (King's Score), Goteborg University Cirrhosis Index (GUCI), FibroIndex, and FornsIndex, and determine their accuracy in predicting bleeding events in hepatitis B liver cirrhosis patients. Simultaneously, we compared the predictive performance of transient elastography.

## Methods

### Study Patients

This was a prospective cohort study, and consecutive hospitalized patients with hepatitis B liver cirrhosis were admitted to the Department of Gastroenterology, the First Affiliated Hospital of Nanchang University in China, between February 2017 and January 2021. The patient inclusion criteria were as follows: (1) age ≥ 18, (2) diagnosis of hepatitis B cirrhosis (positive hepatitis B surface antigen, and diagnosed with cirrhosis by liver biopsy or imaging examinations together with clinical features such as ascites, thrombocytopenia or gastro-esophageal varices), (3) first bleeding in the past and received secondary prevention of variceal rebleeding (endoscopic variceal ligation (EVL) combined with a non-selective beta-blocker (NSBB) or EVL alone), and (4) had a liver transient elastography measurement before the second episode of variceal bleeding, The exclusion criteria included the following: (1) a diagnosis of HCC at inclusion or during the first 6 months of follow-up, (2) known HIV, (3) the first bleeding is non-esophagogastric vein bleeding under digestive endoscopy, (4) history of liver transplantation, (5) combination with other types of liver disease such as alcoholic cirrhosis or hepatitis C cirrhosis, (6) the patient had a large amount of ascites at the time of admission or a status of Child–Pugh C class, and (7) severe heart and lung disease. The treatment of the included patients was individualized according to Baveno VI standards. The study protocol was approved by the institutional ethics committee of the First Affiliated Hospital of Nanchang University (No. 2015–1206). Informed written consent was obtained from all the study participants.

### Clinical Data Collection and Follow-Up

Clinical data such as age, sex, diabetes, hypertension, etiology, white blood cell (WBC), hemoglobin (HB), platelet (PLT), alanine aminotransferase (ALT), aspartate aminotransferase (AST), total bilirubin (TBIL), albumin (ALB), gamma-glutamyl transpeptidase (γ-GT), alkaline phosphatase (ALP), creatinine (Cr), international normalized ratio (INR), prothrombin time (PT), fibrinogen, blood urea nitrogen (BUN), HBV DNA, hepatitis B e antigen (HBeAg), and liver stiffness measurements were collected at the time of the first acute variceal bleeding. The Child–Pugh score and model for end-stage liver disease (MELD) score were also recorded. The data were collected independently by two physicians and checked by a third person. All included patients were followed up for rebleeding and survival. The primary outcome was a rebleeding event due to esophageal varices.

### Liver Stiffness Measurements and Calculation of Scores

The liver stiffness measurements were completed within 1 week after the patient underwent ligation for acute bleeding. For patients with ascites at the time of admission, the LSM was measured after the ascites subsided. Transient elastography was performed with FibroScan (Echosens, Paris, France) using the standard-probe, and on a fasting (4 h) patient lying flat on his/her back, with the right arm tucked behind the head to facilitate access to the right upper quadrant. The probe is positioned perpendicular to the skin surface in one of the intercostal spaces adjacent to the right lobe of the liver. LSM was considered reliable only if 10 successful acquisitions were obtained and the ratio of the interquartile range over the median (IQR/LSM) was ≤ 0.3. LSM was expressed in kilopascals. Patients with unreliable LSM results had the examination repeated immediately; the results were not analyzed if they remained unreliable. The operators were blinded to all clinical data and the diagnoses of the patients.

A total of four non-invasive models were performed for all included patients:

a. APRI: AST (U/L)/upper limit of normal/PLT (10^9^/L) × 100
b. FIB-4: [age (years) × AST (U/L)]/[PLT (10^9^/L) × √ALT (U/L)]
c. King's score: age (years) × AST (U/L) × INR/PLT (10^9^/L)
d. GUCI: [AST (U/L)/upper limit of normal] × [(prothrombin – INR × 100)/PLT (10^9^/L)]
e. Fibrosis index: 8.28 – 0.01 × PLT (10^9^/L)—serum albumin (g/dl)
f. Forns score: 7.811 – 3.131 × ln [PLT (109/L)] + 0.781 × ln [GGT (U/L)] + 3.467 × ln [age (years)] – 0.014 [cholesterol (mg/dl)]
g. MELD: 3.78 × ln [TBil (μmol/L)] + 11.2 × ln (INR) + 9.57 × ln [creatinine (mg/dl)] + 6.43
h. MELD-Na: MELD + 1.59 × [135-Na^+^ (mmol/L)]
i. ALBI: −0.085 × [albumin (g/L)] + 0.66 × lg [TBil (μmol/L)].

### NSBB Treatment and EVL Procedure

For patients receiving NSBB treatment, either carvedilol or propranolol was used. Carvedilol was started at an initial dose of 6.25 mg once daily and adjusted gradually to the maximum tolerated dose, keeping the heart rate at >55 beats per minute and systolic blood pressure at >90 mmHg. Propranolol was started at an initial dose of 10 mg three times daily and adjusted gradually to the maximum tolerated dose, keeping the heart rate at >55 beats per minute and the systolic blood pressure at >90 mmHg. EVL was performed using commercial multiband devices under sedation with propofol. The varices were ligated from the cardia to the oral side.

### Statistical Analysis

Continuous variables are shown as the mean and standard deviation (SD) or median and interquartile range (IQR), while categorical variables are shown as frequencies (%). The rebleeding rate for the study population was generated using the Kaplan–Meier method, and differences in rebleeding rate were examined using the log-rank test. We tested whether the explanatory variable had an interaction and found no significant interactions within the included variables. Student's *t*-tests or Mann–Whitney *U*-test were performed for group comparisons. The diagnostic accuracy of rebleeding was assessed by receiver operating characteristic (ROC) analysis. Areas under the ROC curves (AUROCs) were compared by the method of DeLong et al. A time-dependent ROC curve was employed to evaluate the time-dependent predictive performance of the model to be tested for rebleeding. All levels of significance were set at a two-sided 5% level. All analyses were performed using SPSS 25.0 IBM (IBM Corp., Armonk, NY, USA) and R 3.5.2 (R Project for Statistical Computing, Vienna, Austria). The R statistical packages “pROC,” “survival,” “compareGroups,” and “survminer” were used to calculate the clinical characteristics table, Kaplan–Meier curves, ROC curve, and time-dependent ROC curves.

## Result

**Figure 1:**
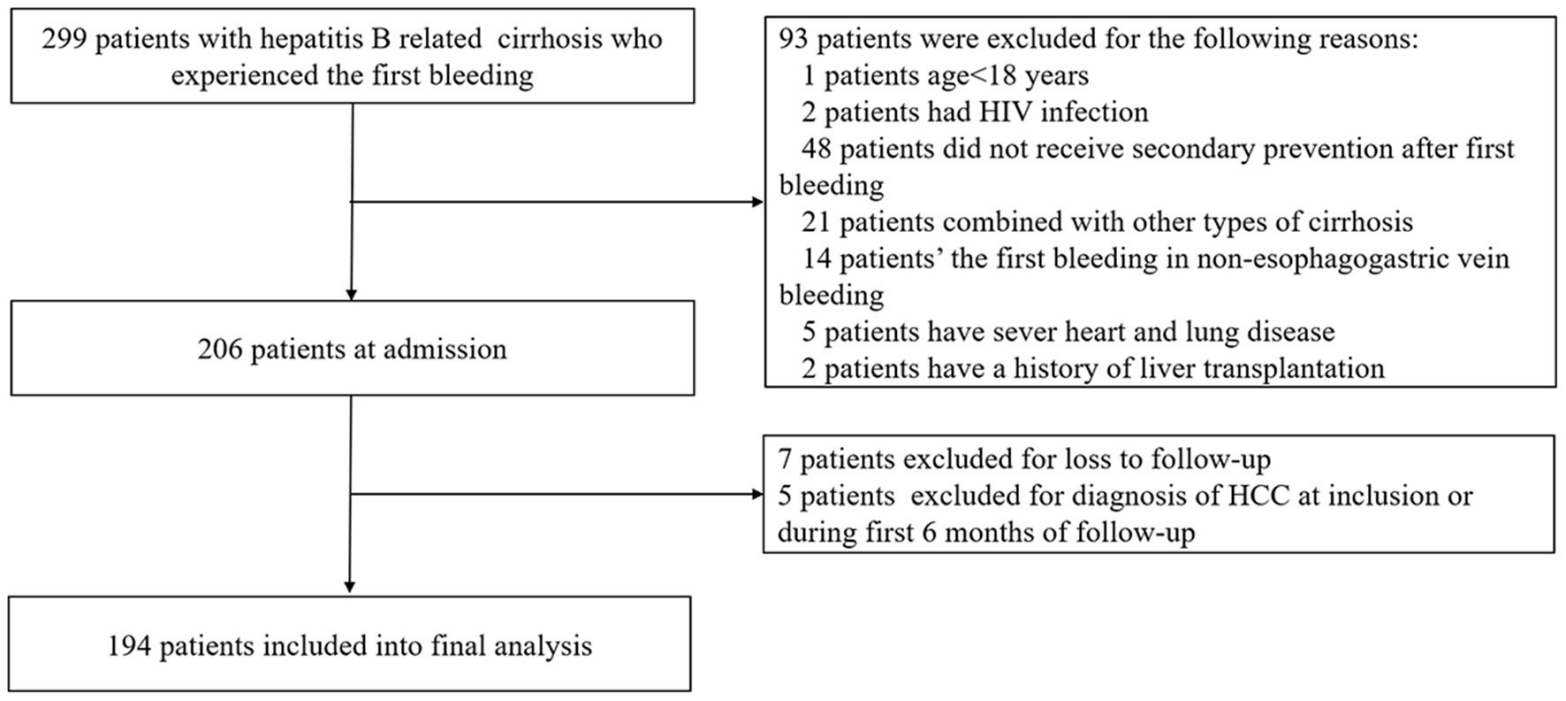
The flowchart of our study.

A total of 299 patients with suspected HBV-related liver cirrhosis underwent series of examinations. Of these, 93 patients were excluded for the exclusion criteria. The remaining 206 patients were followed up. Finally, 194 patients were included in the final analysis. A flow chart for the study enrollment is summarized in Figure 1.

### Patients' Characteristics

The baseline characteristics of the 194 patients with HBV-related liver cirrhosis are shown in Table 1. The average age of the whole population was 52.83 years, and the majority of them were male (81.96%). The baseline median LSM value was 14.57 KPa, and the BMI value was 22.01. The median Child–Pugh, MELD, MELD-Na, and ALBI scores were 6, 9.64, 3.83, and −2.073, respectively. Patients of Child–Pugh class A (86.08%) comprised the majority of this cohort. Various median fibrosis scores were as follows: APRI, FIB-4, King's score, GUCI, FibroIndex, and Forns score were 1.537, 5.029, 32.765, 1.846, 2.588, and 9.254, respectively.

**Table 1:** Baseline characteristics of patients.

**Variable**	**All patients (*N* = 194)**
Male	159 (81.96)
Follow-up time (weeks)	63.28 (17.64–112.5)
Age (years)	52.83(11.43)
BMI	22.01(3.16)
ALT (U/L)	25 (17–40)
AST (U/L)	35 (27–50)
Hemoglobin (g/L)	98.23(29.3)
Platelet count (10^9^/L)	69.5 (47–114)
White Blood Count (10^9^/L)	4.3(2.3)
Albumin (g/L)	34.67 (5.43)
Globulins (g/L)	26.95 (7.89)
Total bilirubin (μmol/L)	20.25 (14–31.9)
INR	1.24 (1.14–1.36)
PT (s)	13.9 (12.7–15)
γ-GT (U/L)	30 (19–59.5)
Cholesterol (mmol/L)	3.18 (2.55–3.86)
Creatinine (μmol/L)	66.4 (57.3–79.1)
Serum sodium (mmol/L)	139.4 (135.8–141.2)
HBV DNA (log10 IU/ml)^*^	3.121(1.701)
HBeAg (positivity rate, %)	33 (17.01)
LSM (kPa)	14.57 (11.22–18.81)
APRI	1.537 (0.807–2.523)
FIB-4	5.029 (2.887–9.495)
King's score	32.765 (17.829–63.320)
GUCI	1.846 (0.942–3.232)
FibroIndex	2.588 (2.242–2.881)
Forns score	9.254 (7.882–10.609)
MELD	9.64 (8.13–11.59)
MELD-Na	3.83 (−1.41 to 9.40)
ALBI	−2.073 (−2.424 to 1.694)
Child–Pugh score	6 (5–7)
Child A	167 (86.08)
Child B	27 (13.92)
Rebleeding events	55 (28.35)
6-week rebleeding event	13 (6.70)
3-month rebleeding events	26 (13.40)
1-year rebleeding events	47 (24.23)

* *For patients with liver cirrhosis who are positive for HBV DNA*.

*BMI, body mass index; ALT, alanine aminotransferase; AST, aspartate aminotransferase; LSM, liver stiffness measurement; PT, prothrombin time; γ-GT, gamma-glutamyl transpeptidase*.

When they were hospitalized due to bleeding esophageal varices for the first time, eight patients developed hepatic encephalopathy below stage III, five patients had fever with bacteremia or spontaneous peritonitis, and one developed hepatorenal syndrome. All patients who suffered complications during hospitalization had fully recovered from the above complications when they were discharged.

Patients were followed up until the occurrence of a rebleeding episode. At a median follow-up of 68.28 weeks (range, 1.0–208.5 weeks), rebleeding occurred in 55 out of 194 patients (28.35%). Among them, the rates of rebleeding within 6 weeks, 3 months, and 1 year were 6.7, 13.4, and 24.23%, respectively. At the same time, three people developed hepatocellular carcinoma during the follow-up period and three people died: two of them from respiratory failure and one from hypovolemic shock during rebleeding.

### Comparison of Baseline Characteristics of Patients With or Without Rebleeding

The comparison of clinical baseline characteristics between the non-rebleeding group and the rebleeding group is shown in Table 2. Some variables in the rebleeding group, such as follow-up time, hemoglobin, platelet count, and white blood count, were smaller than those in the no rebleeding group (*p* < 0.05). However, total bilirubin, INR, APRI, FIB-4, GUCI, FibroIndex, and Forns score were higher in the rebleeding group than in the no rebleeding group (*p* < 0.05).

**Table 2:** Characteristics between the no rebleeding group and rebleeding group.

**Variable**	**No rebleeding** **(*N* = 139)**	**Rebleeding** **(*N* = 55)**	***P*-value**
Follow-up time (weeks)	85.4 (51.9–168)	14.9 (6.43–35.8)	**<0.001**
Age	52.5 (12.1)	53.7 (9.69)	0.461
BMI	22.1 (3.33)	22.1 (2.73)	0.878
ALT (U/L)	25.0 (17.0–38.0)	27.0 (18.5–41.5)	0.243
AST (U/L)	34.0 (27.0–48.0)	35.0 (26.5–55.0)	0.474
Hemoglobin (g/L)	102 (29.5)	88.6 (26.4)	**0.003**
Platelet count (10^9^/L)	82.0 (54.5–134)	54.0 (38.5–77.0)	**<0.001**
White blood count (10^9^/L)	4.56 (2.22)	3.64 (2.24)	**0.011**
Albumin (g/L)	35.0 (5.68)	33.9 (4.71)	0.155
Golbulins (g/L)	27.1 (7.93)	26.5 (7.83)	0.602
Total bilirubin (μmol/L)	19.6 (12.6–30.2)	24.6 (18.2–37.0)	**0.007**
INR	1.22 (1.12–1.35)	1.28 (1.20–1.42)	**0.012**
PT (s)	13.7 (12.6–14.9)	14.2 (13.0–15.2)	0.205
γ-GT	32.0 (19.5–67.5)	25.0 (18.0–47.5)	0.086
Cholesterol (mmol/L)	3.19 (2.63–3.91)	3.12 (2.45–3.56)	0.171
Serum sodium (mmol/L)	139 (136–141)	140 (134–141)	0.587
Creatinine (μmol/L)	66.3 (59.5–78.2)	67.8 (56.1–82.8)	0.883
HBV-DNA			**<0.001**
Negative	77 (55.4%)	6 (10.9%)	
Positive	62 (44.6%)	49 (89.1%)	
NSBB drugs			**<0.001**
no used	36 (25.9%)	31 (56.4%)	
Used	103 (74.1%)	24 (43.6%)	
APRI	1.28 (0.70–2.00)	2.32 (1.15–3.27)	**<0.001**
FIB-4	4.13 (2.53–7.87)	6.77 (4.41–11.8)	**<0.001**
King's score	47.0 (79.0)	95.7 (191)	0.072
GUCI	1.64 (0.86–2.59)	3.01 (1.52–4.26)	**<0.001**
FibroIndex	2.53 (2.17–2.80)	2.71 (2.53–2.97)	**0.001**
Forns score	8.78 (7.30–10.4)	10.2 (9.08–11.0)	**<0.001**
Child–Pugh	6.00 (5.00–7.00)	6.00 (6.00–7.00)	0.118
MELD	9.43 (8.13–11.4)	10.1 (8.67–12.2)	**0.039**
MELD-Na	3.38 (−1.59 to 8.26)	4.89 (0.41–11.2)	0.067
ALBI	−2.20 (−2.45 to −1.74)	−1.87 (−2.20 to −1.63)	**0.012**
LSM (kPa)	12.9 (10.5–15.7)	20.0 (17.9–23.0)	**<0.001**

*BMI, body mass index; ALT, alanine aminotransferase; AST, aspartate aminotransferase; LSM, liver stiffness measurement; PT, prothrombin time; γ-GT, gamma-glutamyl transpeptidase*.

*Data are given as mean ± standard deviation, median (interquartile range), or as percentage of cases (%)*.

*The bold values indicates that these variables are significantly different and specific details has been explained in the Methods - Statistical analysis Section*.

Considering the differences between the NSBB drugs used and HBV-DNA positivity in the two groups of patients, further subgroup analysis was performed. In the two groups of people who used and did not use NSBB, their rebleeding rates were 46.27% (31/67) and 18.89% (24/127), respectively. There was a significant difference in the occurrence of rebleeding events and LSM between the two groups. The remaining clinical features are summarized in Table 3. In this context, the same method was used to compare whether HBV-DNA was positive for patients with liver cirrhosis. Among HBV DNA-negative patients, those who experienced rebleeding and those who did not experience rebleeding, only LSM was significantly different, and the complete comparison is summarized in Table 4.

**Table 3:** Baseline comparison of patients with and without NSBB drugs.

	**No used NSBB drugs**			**Used NSBB**		
	**No rebleeding**	**Rebleeding**	***P*-value**	**No rebleeding**	**Rebleeding**	***P*-value**
	***N* = 36**	***N* = 31**		***N* = 103**	***N* = 24**	
Following-up time (weeks)	64.9 (27.0–167)	11.3 (5.43–30.0)	**<0.001**	87.0 (56.4–160)	19.3 (8.43–46.4)	**<0.001**
Age (years)	55.9 (11.3)	54.2 (10.8)	0.539	51.3 (12.2)	53.1 (8.24)	0.392
BMI	21.9 (3.47)	22.2 (3.31)	0.739	22.2 (3.29)	21.9 (1.77)	0.525
ALT (U/L)	31.0 (24.8–47.0)	28.0 (22.0–47.0)	0.910	23.0 (15.5–34.0)	21.5 (16.0–35.0)	0.753
AST (U/L)	37.5 (32.8–56.8)	42.0 (28.5–53.5)	0.651	32.0 (25.0–41.5)	30.5 (24.0–55.5)	0.121
Hemoglobin (g/L)	113 (33.7)	92.7 (27.9)	**0.009**	98.3 (27.1)	83.3 (23.8)	**0.011**
Platelet count (10^9^/L)	96.5 (51.0–122)	52.0 (34.5–74.0)	**<0.001**	77.0 (55.5–135)	56.5 (41.5–83.8)	**0.009**
White blood count (10^9^/L)	4.85 (1.92)	3.62 (2.57)	**0.034**	4.46 (2.32)	3.65 (1.79)	0.069
Albumin (g/L)	34.3 (6.53)	34.1 (5.29)	0.931	35.2 (5.36)	33.5 (3.93)	0.072
Golbulins (g/L)	28.1 (7.98)	25.6 (8.40)	0.234	26.8 (7.93)	27.6 (7.04)	0.645
Total bilirubin (μmol/L)	25.8 (15.0–39.3)	23.0 (18.4–34.5)	0.602	18.5 (12.3–25.8)	26.4 (17.2–40.1)	**0.015**
INR	1.21 (1.15–1.38)	1.28 (1.23–1.42)	0.178	1.22 (1.12–1.34)	1.28 (1.16–1.39)	0.086
PT (s)	13.8 (12.4–15.4)	14.2 (13.1–14.9)	0.615	13.7 (12.7–14.8)	14.2 (12.9–15.6)	0.294
γ-GT (U/L)	46.0 (24.5–102)	24.0 (16.5–45.5)	**0.012**	30.0 (19.0–56.0)	27.0 (18.0–52.0)	0.714
Cholesterol (mmol/L)	3.37 (2.88–4.11)	3.18 (2.58–3.50)	0.170	3.18 (2.58–3.89)	2.80 (2.35–3.60)	0.253
Serum sodium (mmol/L)	139 (137–141)	140 (134–142)	0.826	139 (136–141)	138 (135–141)	0.407
Creatinine (μmol/L)	64.8 (57.2–78.2)	66.3 (55.1–78.1)	0.651	66.4 (60.5–78.2)	68.7 (60.1–84.2)	0.390
HBV-DNA			**<0.001**			**<0.001**
Negative	20 (55.6%)	3 (9.68%)		57 (55.3%)	3 (12.5%)	
Positive	16 (44.4%)	28 (90.3%)		46 (44.7%)	21 (87.5%)	
APRI	1.59 (0.92–2.24)	2.68 (1.55–3.61)	**0.011**	1.20 (0.65–1.98)	1.88 (1.05–2.56)	**0.037**
FIB-4	4.30 (3.29–8.92)	7.02 (5.42–13.0)	0.061	4.10 (2.44–7.46)	6.25 (3.70–9.34)	0.034
King's score	60.3 (86.9)	88.4 (93.0)	0.208	42.3 (76.0)	105 (272)	0.275
GUCI	2.03 (0.96–3.30)	3.21 (1.90–4.72)	**0.010**	1.52 (0.79–2.42)	2.16 (1.39–3.77)	**0.025**
FibroIndex	2.61 (2.23–2.94)	2.71 (2.50–3.02)	0.200	2.50 (2.15–2.75)	2.72 (2.53–2.92)	**0.004**
Forns score	8.73 (7.88–10.4)	10.2 (9.18–11.6)	**0.012**	8.78 (7.02–10.3)	10.1 (8.44–10.7)	**0.025**
Child–Pugh	6.00 (5.75–7.25)	6.00 (6.00–7.00)	0.698	6.00 (5.00–7.00)	6.00 (6.00–8.00)	0.080
MELD	9.16 (8.13–11.4)	10.1 (8.56–11.8)	0.187	9.45 (8.13–11.4)	10.1 (8.77–13.0)	0.151
MELD-Na	2.94 (−1.84 to 7.56)	4.50 (−0.79 to 11.7)	0.155	3.41 (−1.57 to 8.26)	6.04 (1.44–10.3)	0.158
ALBI	−2.14 (−2.43 to −1.64)	−1.83 (−2.20 to −1.63)	0.386	−2.23 (−2.49 to −1.77)	−1.93 (−2.17 to −1.67)	**0.025**
LSM (kPa)	13.6 (11.0–16.4)	20.1 (17.9–22.4)	**<0.001**	12.5 (10.5–15.3)	19.2 (16.6–23.4)	**<0.001**

*BMI, body mass index; ALT, alanine aminotransferase; AST, aspartate aminotransferase; LSM, liver stiffness measurement; PT, prothrombin time; γ-GT, gamma-glutamyl transpeptidase*.

*Data are given as mean ± standard deviation, median (interquartile range), or as percentage of cases (%)*.

*The bold values indicates that these variables are significantly different and specific details has been explained in the Methods - Statistical analysis Section*.

**Table 4:** Baseline comparison of patients with and without HBV-DNA positivity.

	**HBV-DNA (–)**			**HBV-DNA (+)**		
	**No rebleeding**	**Rebleeding**	***P*-value**	**No rebleeding**	**Rebleeding**	***P*-value**
	***N* = 77**	***N* = 6**		***N* = 62**	***N* = 49**	
Follow-up time (weeks)	76.3 (40.3–145)	40.1 (21.5–68.1)	0.077	88.7 (57.4–172)	12.1 (6.43–30.0)	**<0.001**
Age (years)	53.1 (12.6)	59.0 (11.1)	0.261	51.7 (11.5)	53.1 (9.42)	0.492
BMI	22.0 (3.24)	21.5 (1.47)	0.449	22.3 (3.45)	22.1 (2.85)	0.830
ALT (U/L)	23.0 (15.0–31.0)	25.5 (16.8–32.8)	0.666	27.0 (19.5–47.0)	27.0 (19.0–44.0)	0.891
AST (U/L)	32.0 (24.0–40.0)	28.0 (27.0–35.0)	0.647	36.0 (30.0–53.0)	39.0 (26.0–55.0)	0.677
Hemoglobin (g/L)	99.6 (28.9)	74.0 (27.7)	0.074	105 (30.3)	90.4 (25.9)	**0.007**
Platelet count (10^9^/L)	82.0 (52.0–138)	64.5 (36.2–78.5)	0.152	80.5 (55.5–122)	54.0 (39.0–71.0)	**<0.001**
White blood cell count (10^9^/L)	4.59 (2.50)	3.92 (2.33)	0.526	4.53 (1.84)	3.60 (2.26)	**0.022**
Albumin (g/L)	35.0 (4.78)	32.1 (5.66)	0.266	34.9 (6.67)	34.1 (4.61)	0.417
Globulins (g/L)	27.2 (7.31)	24.8 (9.84)	0.579	27.0 (8.70)	26.7 (7.65)	0.824
Total bilirubin (μmol/L)	20.3 (13.8–31.0)	18.6 (18.3–26.1)	0.799	18.2 (12.3–28.8)	24.8 (18.2–38.8)	**0.007**
INR	1.20 (1.12–1.33)	1.31 (1.23–1.65)	0.124	1.25 (1.14–1.36)	1.28 (1.19–1.41)	0.248
PT (s)	13.4 (12.6–15.0)	14.8 (13.8–18.3)	0.142	13.9 (12.7–14.8)	14.2 (12.9–14.8)	0.605
γ-GT (U/L)	31.0 (18.0–73.0)	35.0 (19.5–50.5)	0.979	34.0 (22.2–60.5)	25.0 (18.0–46.0)	**0.040**
Cholesterol (mmol/L)	3.18 (2.61–3.91)	2.83 (2.23–3.29)	0.308	3.20 (2.70–3.88)	3.12 (2.47–3.57)	0.362
Serum sodium (mmol/L)	139 (136–141)	138 (134–141)	0.752	140 (136–142)	140 (135–141)	0.318
Creatinine (μmol/L)	66.5 (58.4–78.0)	67.2 (50.7–70.5)	0.712	65.8 (59.8–78.6)	67.8 (56.4–83.0)	0.873
NSBB drugs			0.340			**0.002**
No used NSBB	20 (26.0%)	3 (50.0%)		16 (25.8%)	28 (57.1%)	
Used NSBB	57 (74.0%)	3 (50.0%)		46 (74.2%)	21 (42.9%)	
ALBI	−2.16 (−2.44 to −1.84)	−1.77 (−2.23 to −1.41)	0.225	−2.22 (−2.54 to −1.68)	−1.88 (−2.17 to −1.64)	0.077
APRI	1.19 (0.68–1.84)	1.99 (0.91–3.01)	0.268	1.60 (0.84–2.35)	2.32 (1.16–3.33)	**0.011**
FIB-4	4.10 (2.38–7.60)	7.16 (3.71–13.3)	0.149	4.19 (3.00–8.58)	6.77 (4.51–11.8)	**0.010**
King's score	35.4 (33.7)	57.2 (38.6)	0.231	61.4 (111)	100 (202)	0.228
GUCI	1.40 (0.83–2.26)	3.07 (1.18–4.22)	0.176	1.95 (0.94–3.15)	3.01 (1.64–4.29)	**0.015**
FibroIndex	2.53 (2.15–2.73)	2.63 (2.50–2.89)	0.493	2.53 (2.18–2.87)	2.72 (2.53–2.97)	**0.010**
Forns score	8.68 (7.14–10.5)	10.1 (9.75–11.7)	0.088	8.99 (7.65–10.3)	10.2 (9.01–10.9)	**0.003**
Child–Pugh	6.00 (5.00–7.00)	7.00 (5.50–7.75)	0.287	6.00 (5.00–7.00)	6.00 (6.00–7.00)	0.431
MELD	8.72 (6.96–10.7)	12.5 (8.36–16.6)	0.128	10.4 (8.32–12.4)	10.1 (8.72–12.1)	0.896
MELD-Na	3.41 (−1.48 to 7.28)	8.09 (5.58 to 16.4)	0.073	3.13 (−1.61 to 9.31)	4.50 (0.33 to 10.7)	0.297
LSM (kPa)	12.5 (9.96–15.3)	27.5 (22.9–30.7)	**0.001**	13.6 (10.9–16.1)	19.4 (17.8–21.9)	**<0.001**

*BMI, body mass index; ALT, alanine aminotransferase; AST, aspartate aminotransferase; LSM, liver stiffness measurement; PT, prothrombin time; γ-GT, gamma-glutamyl transpeptidase*.

*Data are given as mean ± standard deviation, median (interquartile range), or as percentage of cases (%)*.

*The bold values indicates that these variables are significantly different and specific details has been explained in the Methods - Statistical analysis Section*.

**Figure 2:**
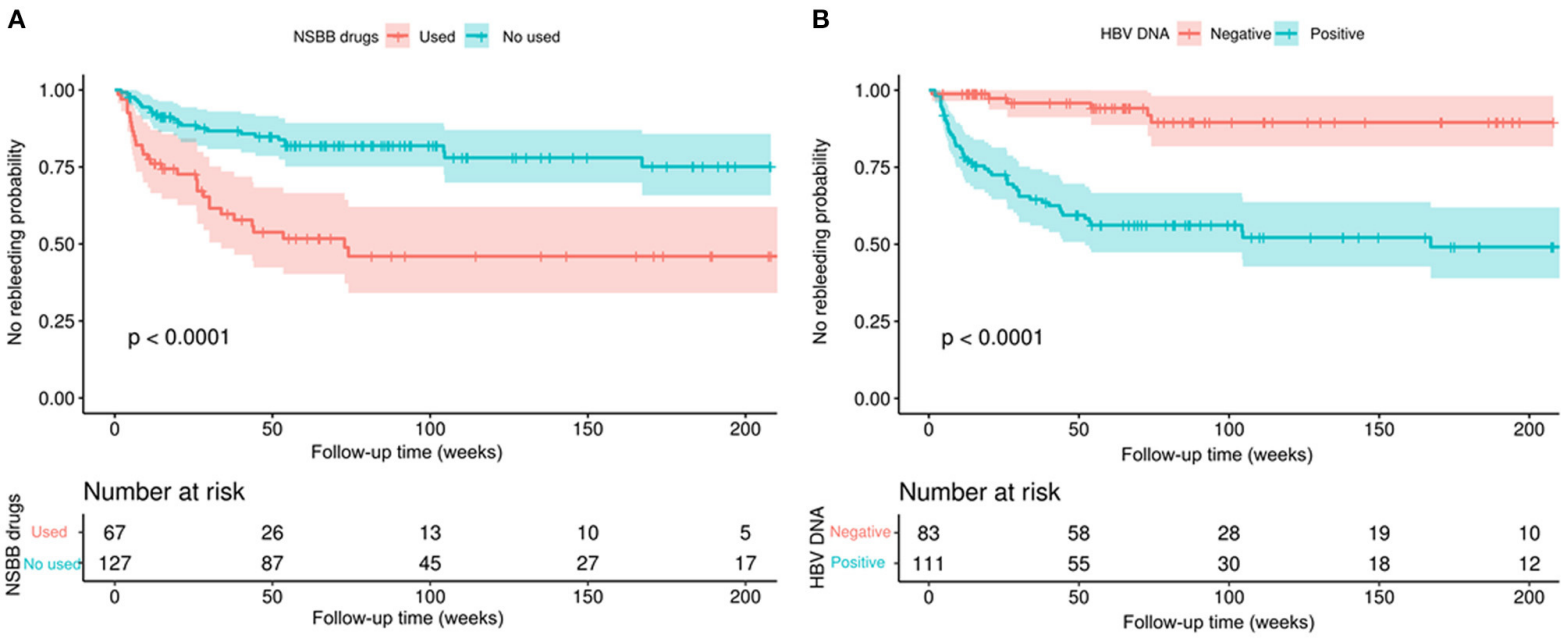
Kaplan–Meier curves demonstrating rebleeding in patients with HBV-related cirrhosis according to different clinical characteristics. **(A)** According to whether NSBB drugs were used to compare the no rebleeding probability between the two groups. **(B)** According to whether HBV-DNA positivity was used to compare the no rebleeding probability between the two groups. *p*-values are from the log-rank test.

According to the two parameters of NSBB drugs and HBV-DNA positivity, two subgroups were divided, and then survival analysis of the two subgroups was performed while drawing Kaplan–Meier curves (Figure 2). In the above two subgroups, there was a significant difference in the survival probability of the two groups of patients in each subgroup (*p* < 0.0001).

### Comparison of Parameters for Prediction of Rebleeding

As shown in Table 5, the area under the ROC curve (AUC) of each parameter at 6 weeks, 3 months, and 1 year was compared. At 6 weeks, 3 months, and 1 year, the maximum and minimum AUC parameters used to predict rebleeding events were LSM (AUC: 0.698) and FibroIndex (AUC: 0.549); LSM (AUC: 0.732) and MELD (AUC: 0.522); and LSM (AUC: 0.735) and MELD-Na (AUC:0.574), respectively. At the above three time points, the AUC of LSM was always the largest (*p* < 0.01). In contrast, the AUC of MELD-Na was <0.6 (*p* > 0.05).

**Table 5:** The predictive value of various parameters at different time points.

	**Variable**	**AUROC**	***P*-value**	**Cut-off point**	**Sensitivity (%)**	**Specificity (%)**
6 weeks	Child–Pugh	0.629 (0.557–0.697)	0.0834	6	66.67	67.6
	MELD	0.582 (0.510–0.653)	0.2968	12.91	40	87.15
	MELD-Na	0.569 (0.496–0.640)	0.4259	6.853	53.33	69.83
	ALBI	0.656 (0.585–0.723)	0.0696	−1.889	73.33	64.25
	APRI	0.624 (0.552–0.692)	0.1417	2.295	60	73.18
	FIB-4	0.622 (0.549–0.690)	0.0964	5.623	66.67	55.87
	King's score	0.632 (0.560–0.700)	0.085	51.694	53.33	71.51
	GUCI	0.632 (0.560–0.700)	0.1049	2.915	60	72.07
	FibroIndex	0.549 (0.476–0.621)	0.5362	2.957	33.33	81.01
	Forns score	0.657 (0.586–0.724)	0.0225	8.999	86.67	48.04
	LSM	0.698 (0.628–0.762)	0.0072	17.77	66.67	72.63
3 months	Child–Pugh	0.583 (0.510–0.653)	0.1034	6	50	68.12
	MELD	0.522 (0.449–0.594)	0.6838	8.78	52.94	37.5
	MELD-Na	0.523 (0.451–0.595)	0.6732	5.282	67.65	43.13
	ALBI	0.561 (0.488–0.632)	0.2752	−1.734	41.18	76.25
	APRI	0.652 (0.581–0.719)	0.0024	2	58.82	71.25
	FIB-4	0.628 (0.555–0.696)	0.0085	5.623	64.71	58.13
	King's score	0.630 (0.558–0.698)	0.0071	14.796	100	23.75
	GUCI	0.646 (0.574–0.713)	0.0029	2.915	52.94	74.37
	FibroIndex	0.607 (0.534–0.676)	0.033	2.628	64.71	57.5
	Forns score	0.613 (0.541–0.682)	0.0236	8.515	82.35	40.63
	LSM	0.732 (0.664–0.793)	<0.0001	17.77	67.65	77.50
1 year	Child–Pugh	0.618 (0.546–0.687)	0.0026	6	45.12	72.32
	MELD	0.604 (0.532–0.674)	0.0108	10.36	50	69.64
	MELD-Na	0.574 (0.501–0.644)	0.0822	3.849	58.54	58.04
	ALBI	0.649 (0.578–0.716)	0.0002	−2.045	64.63	64.29
	APRI	0.686 (0.616–0.751)	<0.0001	2	53.66	80.36
	FIB-4	0.666 (0.595–0.732)	<0.0001	5.554	63.41	65.18
	King's score	0.695 (0.625–0.759)	<0.0001	28.432	75.61	55.36
	GUCI	0.693 (0.623–0.757)	<0.0001	2.62	53.66	82.14
	FibroIndex	0.725 (0.656–0.786)	<0.0001	2.62376	70.73	69.64
	Forns score	0.675 (0.604–0.740)	<0.0001	9.133	70.73	59.82
	LSM	0.735 (0.667–0.796)	<0.0001	14.61	73.17	68.75

*AUROC, Area under receiver operating characteristic*.

**Figure 3:**
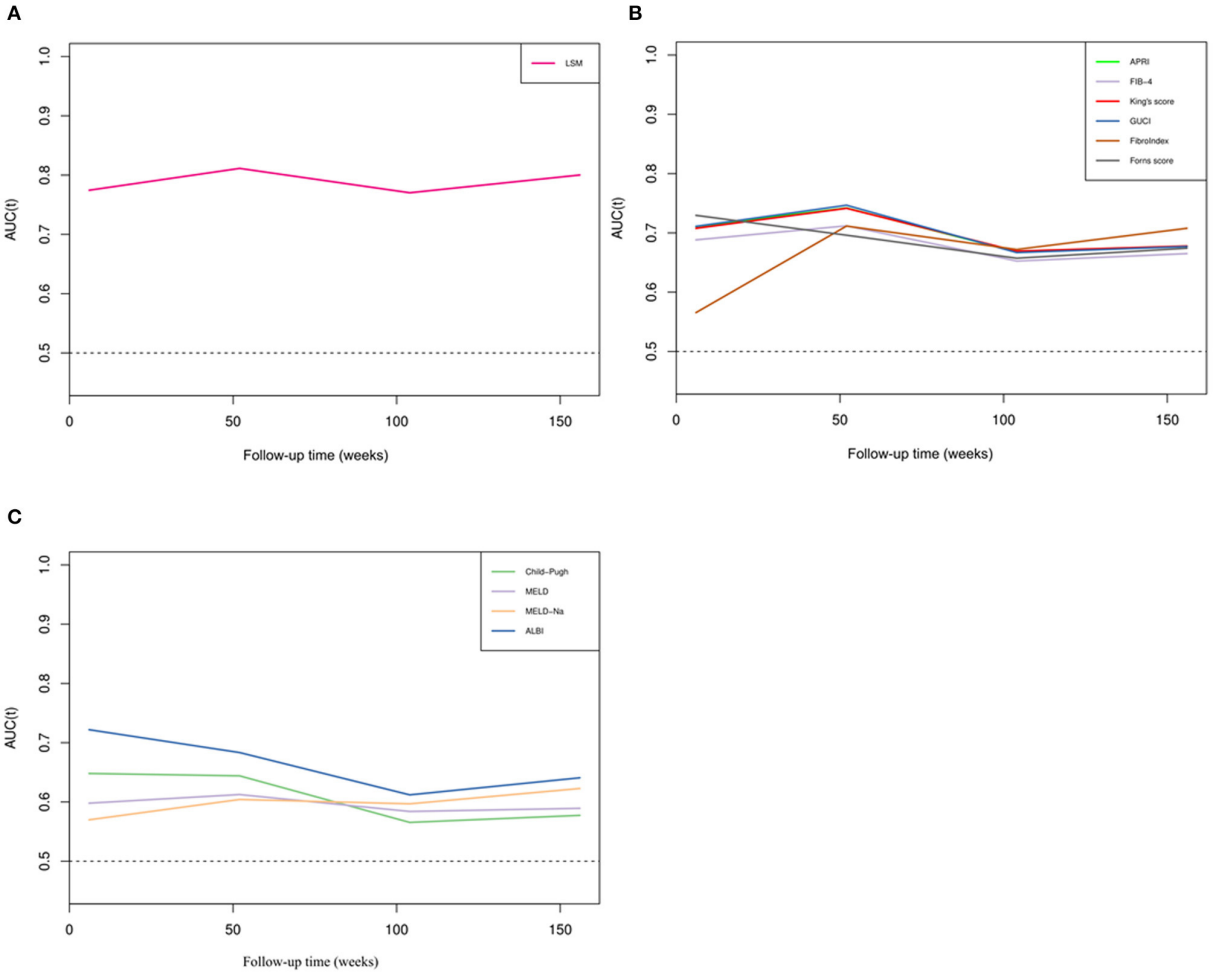
Time-dependent ROC curves of different parameters. **(A)** Time-dependent ROC curve of LSM. **(B)** Time-dependent ROC curves of non-invasive fibrosis scores. **(C)** Time-dependent ROC curves of liver function scores.

To further illustrate the changes in the AUCs of various parameters over time, we drew a time-dependent ROC curve. This curve was simple and represented a method to visually understand the AUC values corresponding to different time points. As shown in Figure 3, various parameters were divided into three categories: the non-invasive fibrosis score-related group (such as APRI, FIB-4, King's Score, GUCI, FibroIndex, FornsIndex), liver function-related score group (such as Child–Pugh, MELD, MELD-Na, ALBI), and LSM. During the follow-up period, it was found that the AUC of LSM remained relatively stable, and the value was high. At this time, the AUC of LSM was 0.837, and the cut-off value was 17.79 kPa. However, the APRI, King and GUCI time-dependent ROC curves almost fit, which means that they had similar predictive capabilities. ALBI performed best in the liver function-related score group, but over time, the AUC predicting rebleeding gradually decreased.

### Prediction of Rebleeding When Combining the Non-invasive Fibrosis Score and LSM

**Figure 4:**
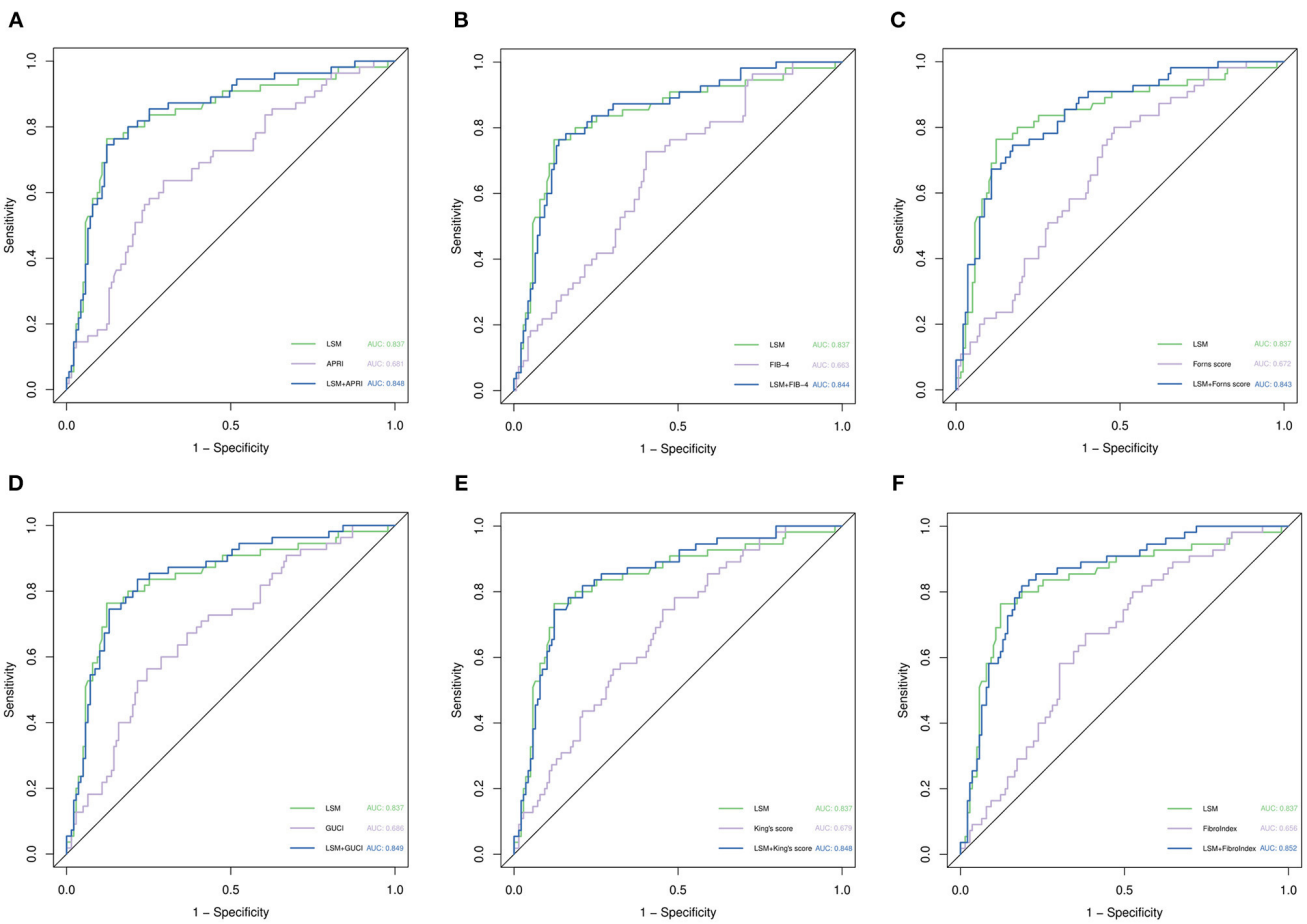
ROC curve analysis for predicting rebleeding by various parameters. **(A)** Comparison of LSM, APRI, and their combined ROC curves. **(B)** Comparison of LSM, FIB-4, and their combined ROC curves. **(C)** Comparison of LSM, Forns score, and their combined ROC curves. **(D)** Comparison of LSM, GUCI, and their combined ROC curves. **(E)** Comparison of LSM, King's score, and their combined ROC curves. **(F)** Comparison of LSM, Fibrosis index, and their combined ROC curves. *p*-values are from the DeLong test (41).

We combined APRI, FIB-4, King's Score, GUCI, FibroIndex, FornsIndex, and LSM and then drew ROC curves separately (Figure 4). Compared with the ROC curve of the non-invasive fibrosis score before the combined diagnosis, the ROC curve obtained after the combined diagnosis was statistically significant (*p* < 0.05), but compared with the ROC curve of LSM, it was not statistically significant (*p* > 0.05). In the entire cohort, the AUCs of LSM and the parameters after combined diagnosis were both over 0.8, suggesting that they have excellent performance in predicting rebleeding events.

## Discussion

In the present study, we first compared the different approaches that use non-invasive tools to predict esophageal venous rebleeding in HBV-related liver cirrhosis, and we found that baseline LSM accurately predicts rebleeding events in our cohort, while different clinical characteristics of patients, such as the use of NSBB drugs and HBV DNA positivity can affect the prognosis of patients. Furthermore, when predicting the occurrence of rebleeding events in the entire cohort, the AUC of LSM reached 0.837 (0.777–0.886), and the cut-off point was 17.79 kPa. At this time, the sensitivity and specificity were 76.36 and 87.77%, respectively, which means that LSM showed excellent performance compared with the non-invasive fibrosis score.

The formation and appearance of varices are driven by various factors, increased portal pressure, collateral blood flow, and vascular endothelial growth factor, all of which contribute to variceal bleeding (1). Spontaneous portosystemic shunting due to portal hypertension is seen in patients with cirrhosis, and it may predict a poor clinical outcome (19, 20). The hepatic venous pressure gradient (HVPG) directly reflects portal hypertension and is a reliable predictor of bleeding due to esophageal varices (21). However, measuring HVPG is an invasive operation that is expensive and requires highly skilled operators. In this case, transient elastography, a non-invasive tool that can indirectly reflect the pathological staging of liver fibrosis and the degree of HVPG, has been widely verified (22, 23). Throughout our follow-up process, LSM predicted the AUC of total rebleeding events was 0.837, which is an exciting result, indicating that this parameter has excellent predictive performance. Indeed, LSM also demonstrated excellent performance in another study on non-alcoholic fatty liver disease (NAFLD) and primary biliary cirrhosis (PBC) (24–26). However, the clinical outcome of these studies focused on death or liver-related events, rather than a certain type of complication, such as recurrent bleeding from cirrhosis. Our research verifies that LSM can reliably predict rebleeding events in hepatitis B-related cirrhosis and further expands the spectrum of diseases in which LSM can be used to predict rebleeding.

Numerous attempts have been made to develop non-invasive fibrosis scores to evaluate the degree of hepatitis B-related liver fibrosis, and these non-invasive fibrosis scores are highly accurate in diagnosing liver fibrosis grading (27), which indirectly reflects the degree of portal hypertension. In our study, six non-invasive fibrosis models were included. Among them, GUCI had the largest AUC (0.686) for predicting rebleed events during the entire follow-up episode, which was similar to the AUC of APRI (0.681). The end point of our study was different, constituting the event of clinical rebleeding, not the pathological stage of liver fibrosis, so the area under the ROC curve we calculated was small. Indeed, a study evaluating the prognostic effect of non-invasive fibrosis scores in patients with non-alcoholic fatty liver disease has pointed out that these scores can help identify patients with NAFLD who are at increased risk for liver-related complications or death (6). The main connection between the above research and this research is to propose that the non-invasive fibrosis score can be related to the prognosis of patients with liver disease rather than just reflecting the degree of liver fibrosis. However, the accuracy of the non-invasive fibrosis score in predicting rebleeding events is not as high as that of LSM. The reason can be found in another article describing LSM as superior to non-invasive fibrosis in the staging of liver fibrosis (28). We tried to combine LSM and non-invasive fibrosis scores to predict rebleeding events, but the combined model did not significantly improve the diagnostic performance. Some studies have confirmed that the abovementioned combined model can improve the accuracy of diagnosing liver fibrosis and avoid liver biopsy (29, 30). In addition to TE, two-dimensional shear wave elastography is a promising marker in predicting esophageal varices and portal hypertension with high accuracy (31–33).

Interestingly, when we drew the time-dependent ROC curve, the scores curves, such as those for the APRI, King's score, and GUCI, almost fit, and their AUCs were also similar. These three scores were included in the two variables of AST and PLT. In our entire cohort, the variation range of AST was not as large as that of PLT, so when calculating the score, the weight of PLT change was high. On the other hand, these three scores showed similar performance in diagnosing liver fibrosis (10, 27), which can also give such results.

In our cohort, we included the scores that reflect the functional status of the liver in the analysis and found that ALBI had the highest accuracy among these scores. Furthermore, our results are consistent with previous studies (34), and ALBI is not just a prognostic score for primary hepatocellular carcinoma. However, the predictive performance of ALBI was lower than that of LSM in our study, and its performance in predicting rebleeding events was lower than that in predicting survival (35). In fact, a study suggested that the ALBI score can reflect liver fibrosis and portal pressure in cirrhosis (36). Considering that the patients we included were Child grade A or B, the subjectivity and ceiling effect of the Child score were amplified at this time, and the change spectrum of MELD and MELD-Na was not large, making these predictions less accurate than the ALBI score.

Antiviral drugs and the NSBB drugs are the first-line treatments recommended by the guidelines (12). Among the people who meet the criteria for the guidelines, the incidence of rebleeding events is significantly lower than that of those who do not meet the recommended guidelines (37). Treatment with oral antiviral drugs in patients with HBV cirrhosis is effective in improving liver function and survival in decompensated cirrhosis, and several studies have demonstrated that they are able to reverse liver fibrosis (38). We also found that patients who used NSBB had a lower rebleeding rate than those who did not. This effect can be interpreted as reducing portal blood flow and lowering portal pressure. Moreover, NSBB could also reduce overall mortality or rebleeding (39, 40).

The major limitation of this study lies in the potentially limited external validity of the results for different populations and settings. Since our study is based on a single-center cohort in China, the results may need to be verified by international multi-center trials. Another limitation is that our study failed to record the change in LSM during the follow-up process and thus lacked verification of the change in LSM. However, our rigorous study design concluded that the baseline LSM has excellent predictive performance. Finally, most patients were in Child–Pugh class A, suggesting that the number of patients with late decompensated cirrhosis is relatively low. Thus, our findings may not be readily applicable in a population predominantly with advanced decompensated cirrhosis.

In conclusion, our study demonstrates that LSM seems to be a promising parameter for predicting rebleeding in patients with hepatitis B-related cirrhosis. At the same time, compared with six included non-invasive fibrosis scores and four liver function scores, the prediction performance of LSM was significantly reliable. It is worth emphasizing that different clinical characteristics will also affect the prognosis of patients. However, after the combination of LSM and the non-invasive fibrosis score, the performance of predicting rebleeding cannot be improved further. Hence, we do not recommend such time-consuming work in clinical practice.

## Data Availability Statement

The original contributions presented in the study are included in the article/supplementary material, further inquiries can be directed to the corresponding author/s.

## Ethics Statement

The studies involving human participants were reviewed and approved by the Institutional Ethics Committee of the First Affiliated Hospital of Nanchang University (No. 2015-1206). Written informed consent to participate in this study was provided by the participants.

## Author Contributions

LL and YN contributed equally to this study. LL designed and wrote the original draft. YN analyzed the data and wrote the manuscript. YZ and QL collected the data. XZ critically revised the manuscript. All authors contributed to the article and approved the submitted version.

## Conflict of Interest

The authors declare that the research was conducted in the absence of any commercial or financial relationships that could be construed as a potential conflict of interest.

## Publisher's Note

All claims expressed in this article are solely those of the authors and do not necessarily represent those of their affiliated organizations, or those of the publisher, the editors and the reviewers. Any product that may be evaluated in this article, or claim that may be made by its manufacturer, is not guaranteed or endorsed by the publisher.
